# Supplementary material for: Synergistic Adverse Effects of Azithromycin and Hydroxychloroquine on Human Cardiomyocytes at a Clinically Relevant Treatment Duration
Source: Pharmaceuticals (Basel). 2022 Feb 12;15(2):220. doi: 10.3390/ph15020220 (PMC8877825; doi:10.3390/ph15020220)
Supplement: Supplementary file 1 [file pharmaceuticals-15-00220-s001.zip › pharmaceuticals-1584660-supplementary/pharmaceuticals-1584660-supplementary.pdf]

Supplementary information

Video S1. Contractile behavior of iPSC-CM after 3 days of treatment.

Video S2. Contractile behavior of iPSC-CM after 7 days of treatment.

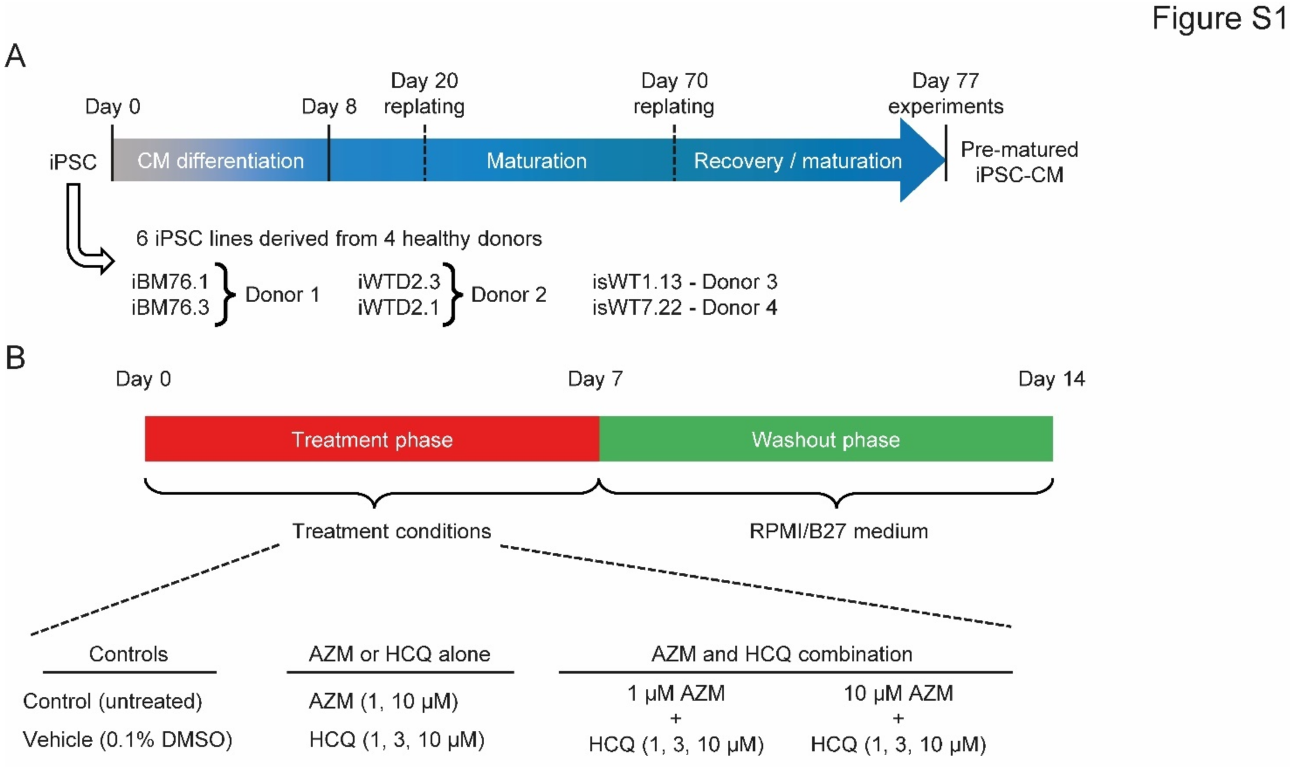

Figure S1. Experimental scheme. (**A**) Timeline of iPSC-CM differentiation and maturation under long-term cultivation for 77 days before the drug treatment and functional analysis. iPSC lines derived from 4 healthy donors were used in the experiments to consider different genetic background. (**B**) Scheme of all conditions used in the study with drug treatment and washout phases.

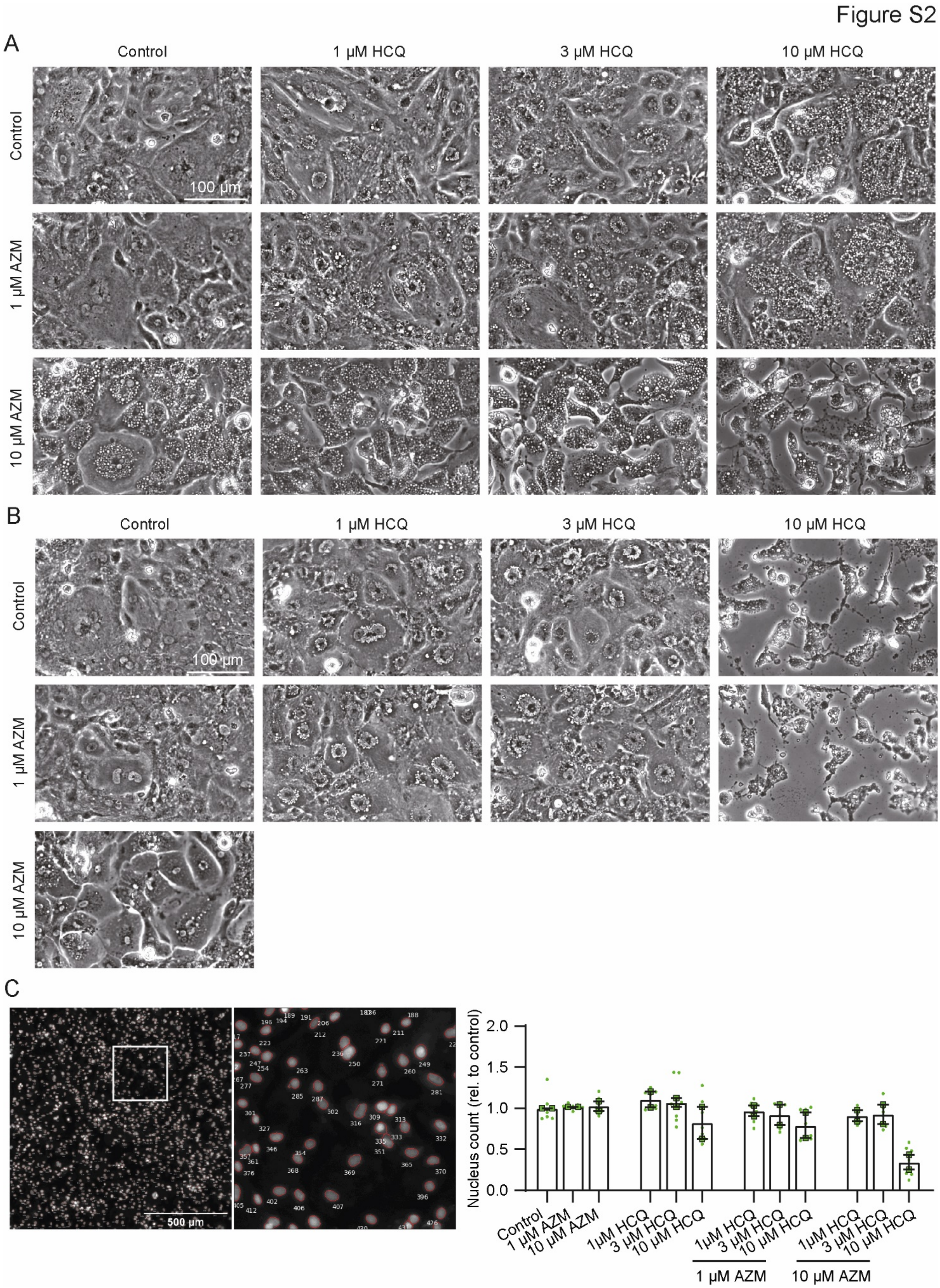

Figure S2. Morphological changes induced by treatment with HCQ and AZM, and quantification of

nucleus number after 7-day treatment with the drugs. (**A, B**) Representative brightfield images depicting morphology of iPSC-CMs after 7-day (**A**) treatments with HCQ and AZM in different concentrations and after subsequent 7-day washout (**B**). Scale bar: 100 µm. (**C**) Representative images of iPSC-CMs stained with Hoechst33342 and nucleus count using CellProfiler software. Nucleus counts after the 7-day drug treatment relative to the control group (n = 7-8 images from 2 independent experiments). Data represent mean and SEM of n = 2 different iPSC-CM differentiations from healthy donor iBM76.

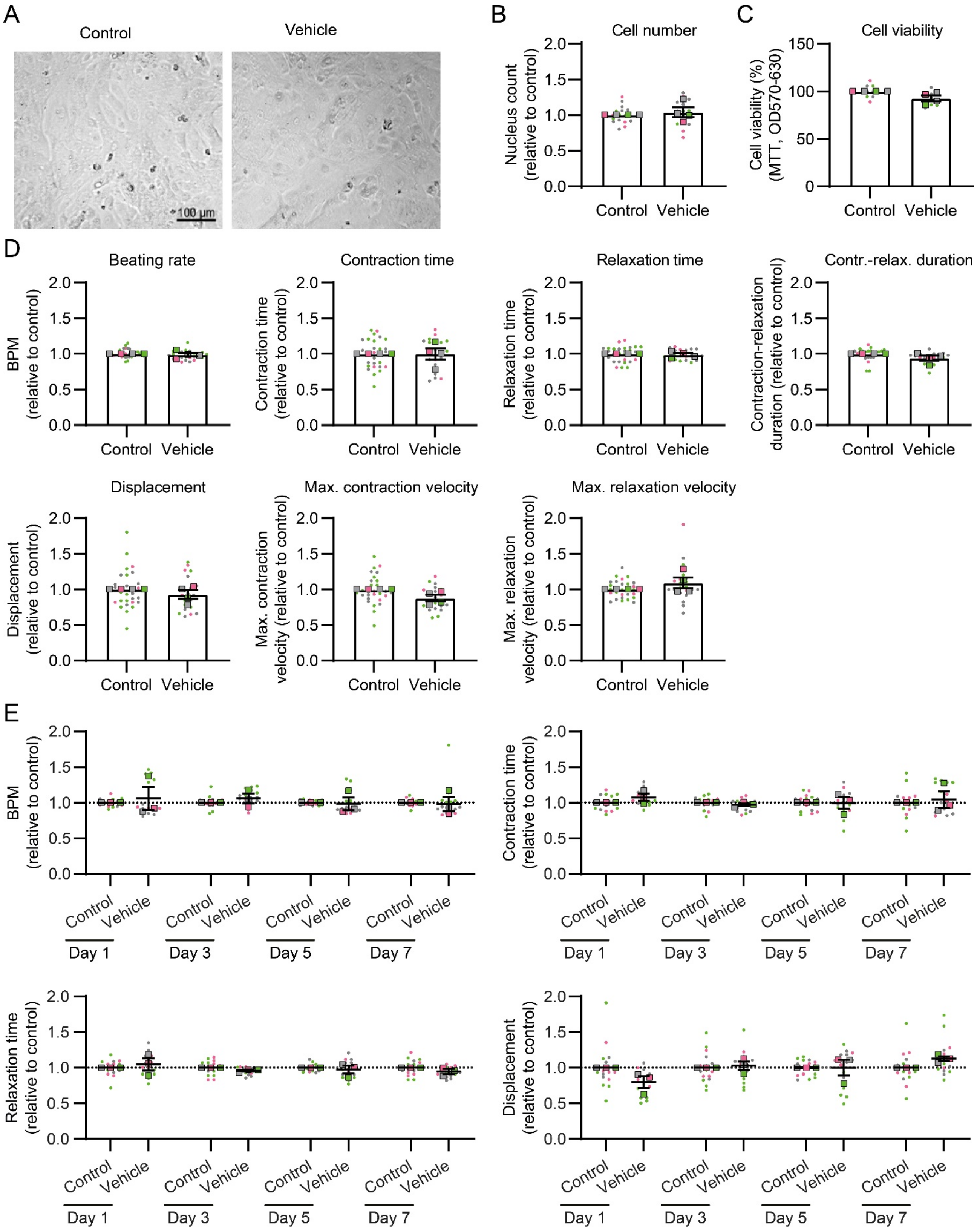

Figure S3. Cell viability and contractility in vehicle (0.1% DMSO) treated iPSC-CMs. (**A**) Brightfield images of iPSC-CMs after 7-day culture with RPMI/B27 medium (control) or in the presence of 0.1% DMSO vehicle showing that vehicle treatment did not affect CM morphology. (**B**) Cell density was

investigated based on the quantification of cell nucleus counts using Hoechst33342 staining and fluorescence imaging. (**C**) Cell viability examined by MTT assay. (**D**) Quantification of contractile function using video-based motion vector analysis. Data represent mean and SEM of n = 4 different iPSC-CM differentiations from 3 healthy donors indicated by different colors (iBM76.3 in green, iWTD2.1 in grey, isWT7.22 in pink). Points show technical replicates and squares represent mean values. (**E**) Changes in beating parameters over time. Pooled individual replicates from n = 3 different iPSC-CM differentiations (iBM76.3 in green, iWTD2.1 in grey, isWT7.22 in pink).

Figure S4

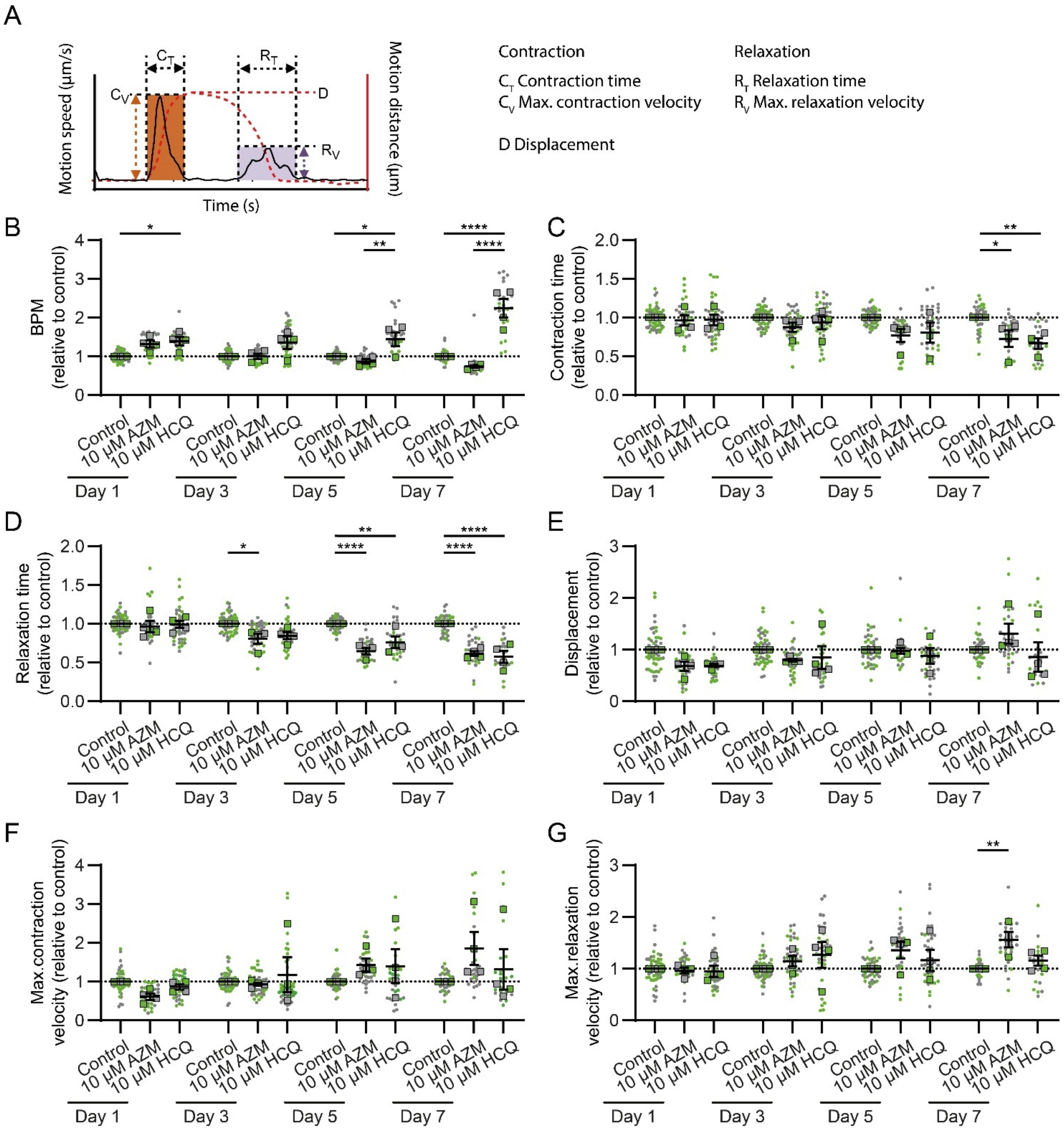

Figure S4. Effects of AZM and HCQ treatments on contraction parameters. (**A**) Scheme of beating trace and calculated parameters using Maia motion analysis software. (**B-G**) Effects of 10 µM AZM and 10 µM HCQ during the treatment period of 7 days. Shown are data of the beating rate (**B**), contraction time (**C**), relaxation time (**D**), displacement (**E**), max. contraction velocity (**F**) and max. relaxation velocity (**G**), which were normalized to the control. Points represent technical replicates and squares depict the means of individual experiments using iPSC-CM from 3 healthy donors indicated by different colors (iBM76.3 in green, iWTD2.1 in grey, isWT7.22 in pink). Lines indicate overall mean. n = 26-54 videos from 4-5 experiments were analyzed per group. Statistical analysis

was performed based on the mean values of the individual experiments using two-way ANOVA and Tukey's multiple comparison test. * $p < 0.05$, ** $p < 0.01$, **** $p < 0.0001$.

Figure S5

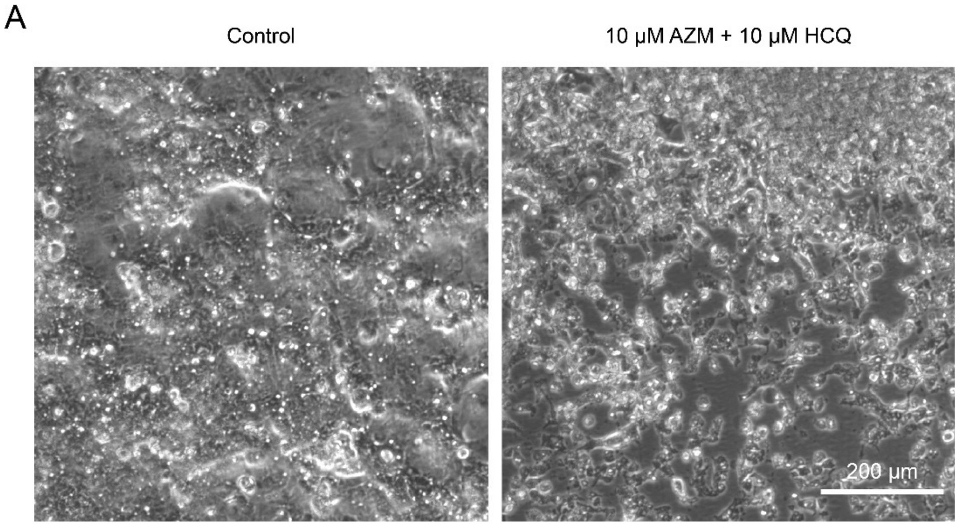

Figure S5. Representative images of iPSC-CMs on MEA plate**.** (**A, B**) Morphology of iPSC-CMs in the vehicle treated (**A**) and 10 µM AZM and 10 µM HCQ treated (**B**) groups at day 8. Combination treatment with 10 µM AZM and 10 µM HCQ increased the number of dead cells.

Figure S6

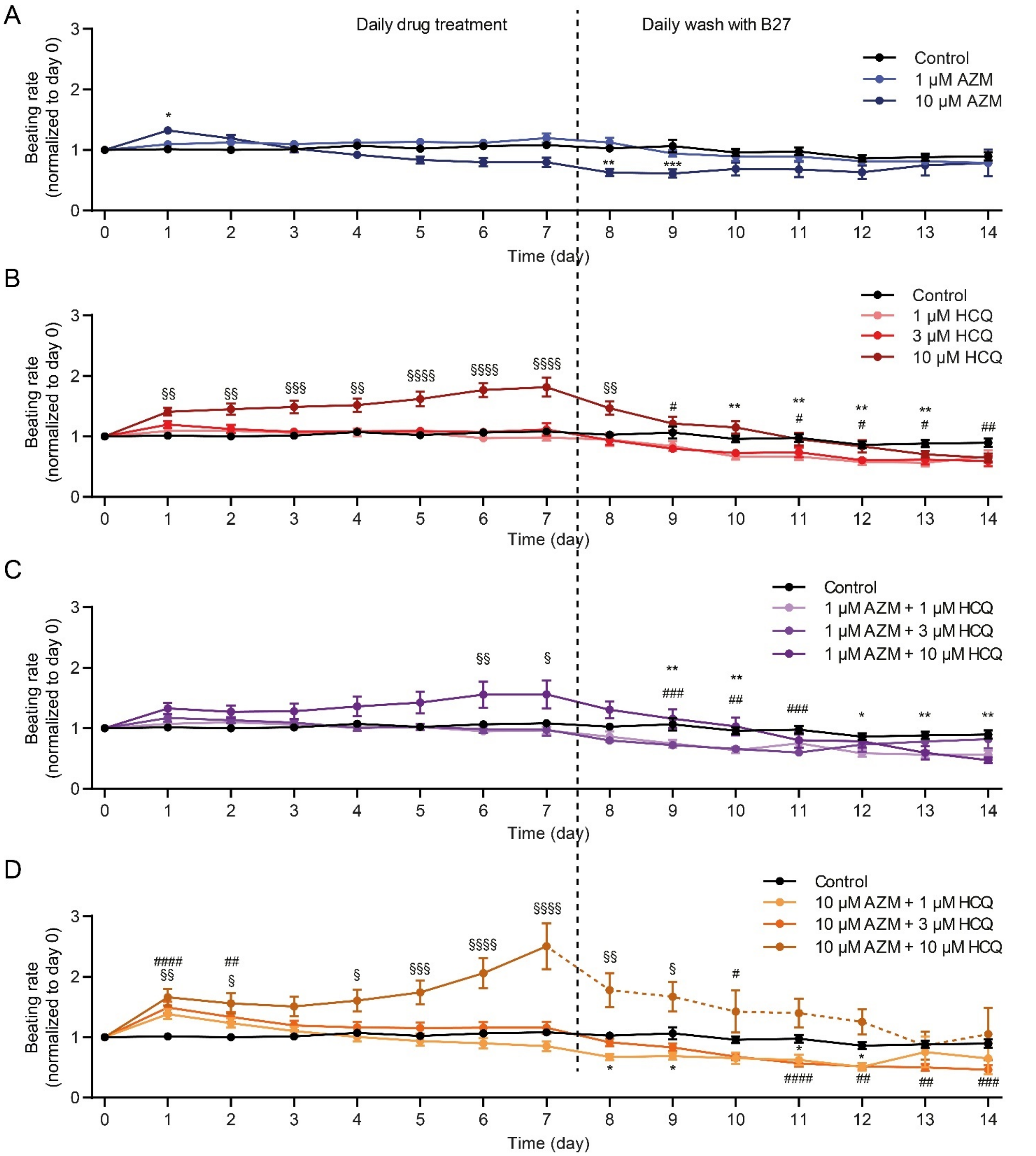

Figure S6. Effects of HCQ and AZM treatments on the beating rates of iPSC-CMs. (**A**, *: 10 μM AZM vs. control) Effect of AZM treatment (1 and 10 μM) on spontaneously beating rates (normalized to day 0) during 7-day drug treatment (left) and following 7-day washout (right). (**B**, *: 1 μM HCQ vs. control, $^{\#}$: 3 μM HCQ vs. control, §: 10 μM HCQ vs. control) Effect of HCQ treatment (1, 3 and 10 μM) on spontaneously beating rates during 7-day drug treatment (left) and following 7-day washout (right). (**C**, *: 1 μM AZM +1 μM HCQ vs. control, $^{\#}$: 1 μM AZM + 3 μM HCQ vs. control, §: 1 μM AZM + 10 μM HCQ vs. control) Effect of HCQ (1, 3, and 10 μM) combined with 1 μM AZM on spontaneous

beating rates during 7-day drug treatment (left) and following 7-day washout (right). (**D**, *: 10 µM AZM +1 µM HCQ vs. control, #: 10 µM AZM + 3 µM HCQ vs. control, §: 10 µM AZM + 10 µM HCQ vs. control) Effect of HCQ (1, 3, and 10 µM) combined with 10 µM AZM on spontaneously beating rates during 7-day drug treatment (left) and following 7-day washout (right). iPSC-CMs derived from four donors were used for MEA recording. For the initial recording (day 0), $10 \leqslant n \leqslant 13$ for all conditions. Numbers of beating iPSC-CM cultures used for the analysis are listed in Supplementary Table 1. Two-way ANOVA with Bonferroni post-hoc test ($^{*,\#,\S}$ $p < 0.05$, $^{**,\#\#,\S\S}$ $p < 0.01$, $^{***,\#\#\#,\S\S\S}$ $p < 0.001$, and $^{\#\#\#\#,\S\S\S}$ $p < 0.0001$).

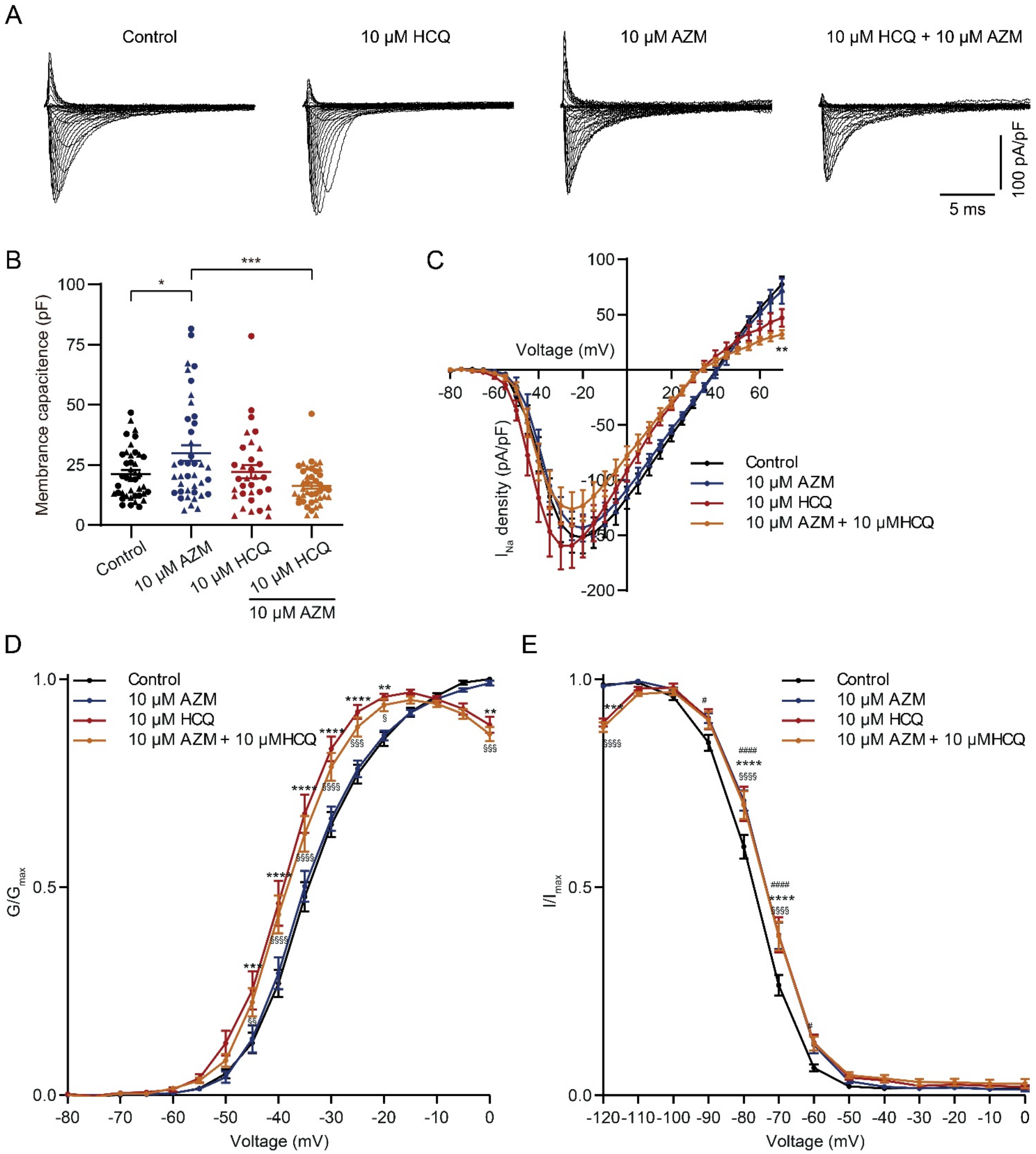

Figure S7. Effects of HCQ and AZM on the sodium channel. (**A**) Representative I_{Na} traces of iPSC-CMs in the control and drug-treated groups (10 μM AZM, 10 μM HCQ, and their combination). (**B**) Statistical analysis of membrane capacitance of iPSC-CMs in the control and drug-treated groups. Different shapes of symbols indicate different differentiations. (**C**, *: 10 μM AZM + 10 μM HCQ vs. control) Statistical analysis of I_{Na} in control and 7-day drug-treated groups. (**D, E**, $^{\#}$: 10 μM AZM vs. control, *: 10 μM HCQ vs. control, §: 10 μM AZM + 10 μM HCQ vs. control) Steady-state activation (**D**) and inactivation (**E**) in control and 7-day drug treated groups. I_{Na} steady-state kinetics are altered

by AZM and HCQ. n=41, 40, 30, and 41 cells for the control, 10 µM AZM-treated, 10 µM HCQ-treated, and AZM and HCQ combination groups, respectively, were analyzed; shown are mean and SEM from 6 independent differentiations (**B-E**). One-way ANOVA with Tukey's multiple comparison test (**B**) and two-way ANOVA with Bonferroni post-hoc test (**C-E**) were used for statistical analysis ($^{*,\#,\S}$ $p < 0.05$, **,§§ $p < 0.01$, ***,§§§ $p < 0.001$, and $^{****,\#\#\#\#,\S\S\S\S}$ $p < 0.001$).

Table S1. Spontaneously beating status of iPSC-CM culture during 15 days of the recording

Number of beating culture	Day 0	Day 1	Day 2	Day 3	Day 4	Day 5	Day 6	Day 7	Day 8	Day 9	Day 10	Day 11	Day 12	Day 13	Day 14
Basal	13	13	13	13	13	13	13	13	13	13	13	13	13	13	13
1 µM AZM	10	10	10	10	10	10	10	10	10	10	10	10	10	10	10
10 µM AZM	11	11	11	11	11	11	11	11	11	11	9	9	9	9	9
1 µM HCQ	10	10	10	10	10	10	10	10	10	10	9	8	8	8	8
3 µM HCQ	10	10	10	10	10	10	10	10	10	10	10	10	9	9	8
10 µM HCQ	12	12	12	12	12	12	12	12	12	12	11	11	10	8	6
1 µM AZM & 1 µM HCQ	10	10	10	10	10	10	10	10	10	10	10	10	9	9	9
1 µM AZM & 3 µM HCQ	11	11	11	11	11	11	11	11	11	11	10	9	8	8	8
1 µM AZM & 10 µM HCQ	11	11	11	11	11	11	11	11	11	11	11	10	8	6	6
10 µM AZM & 1 µM HCQ	11	11	11	11	11	11	11	11	11	11	10	9	9	9	9
10 µM AZM & 3 µM HCQ	11	11	11	11	11	11	11	11	11	11	11	11	8	8	6
10 µM AZM & 10 µM HCQ	11	11	11	11	11	11	11	8	5	4	4	3	2	2	2
